# Supplementary material for: Long-Term Associations between Human Cytomegalovirus Antibody Levels with All-Cause Mortality and Cardiovascular Outcomes in an Australian Community-Based Cohort
Source: Viruses. 2022 Nov 29;14(12):2676. doi: 10.3390/v14122676 (PMC9783113; doi:10.3390/v14122676)
Supplement: Supplementary file 1 [file viruses-14-02676-s001.zip › viruses-2037216-supplementary.pdf]

Table S1. Top 10 leading cause of death in the Busselton Health Study.

Cause of Death	Number (%)
Myocardial infarction	19 (4.29)
Stroke	18 (4.06)
Lung cancer	17 (3.84)
Alzheimer's disease	17 (3.84)
Ischemic heart disease	15 (3.39)
Pancreatic cancer	9 (2.03)
Breast cancer	9 (2.03)
Atherosclerotic heart disease	8 (1.81)
Atrial fibrillation	8 (1.81)
Pneumonia	8 (1.81)

Table S2. Hazard ratios for baseline HCMV antibody levels (log-transformed) in relation to all-cause death, CVD death, ACS, and MACCE at 5- and 10-year follow-ups.

	Model 1[3]		Model 2[4]		Model 3[5]		Model 4[6]	
	HR (95% CI)	p-value	HR (95% CI)	p-value	HR (95% CI)	p-value	HR (95% CI)	p-value
All-cause death								
5 years	1.09 (0.97, 1.22)	0.131	1.07 (0.96, 1.20)	0.211	1.07 (0.96, 1.19)	0.244	1.06 (0.96, 1.18)	0.252
10 years	1.05 (0.99, 1.12)	0.074	1.05 (0.99, 1.11)	0.114	1.04 (0.98, 1.10)	0.162	1.04 (0.98, 1.10)	0.182
CVD death								
5 years	1.05 (0.89, 1.24)	0.570	1.03 (0.88, 1.21)	0.731	1.02 (0.87, 1.20)	0.772	1.03 (0.88, 1.21)	0724
10 years	1.02 (0.92, 1.13)	0.721	0.99 (0.90, 1.10)	0.919	0.99 (0.89, 1.09)	0.817	0.99 (0.89, 1.09)	0.776
ACS								
5 years	0.96 (0.86, 1.07)	0.461	0.95 (0.85, 1.06)	0.388	0.95 (0.85, 1.07)	0.408	0.96 (0.85, 1.07)	0.439
10 years	0.96 (0.90, 1.02)	0.202	0.96 (0.90, 1.02)	0.162	0.96 (0.90, 1.02)	0.198	0.96 (0.90, 1.02)	0.185
MACCE 1[1]								
5 years	1.05 (0.97, 1.14)	0.203	1.04 (0.97, 1.12)	0.289	1.04 (0.97, 1.12)	0.278	1.04 (0.97, 1.12)	0.283
10 years	1.04 (0.99, 1.08)	0.117	1.03 (0.99, 1.08)	0.188	1.03 (0.99, 1.07)	0.196	1.03 (0.98, 1.07)	0.215
MACCE 2[2]								
5 years	1.02 (0.94, 1.11)	0.640	1.01 (0.93, 1.11)	0.735	1.02 (0.93, 1.11)	0.681	1.02 (0.94, 1.11)	0.659
10 years	1.01 (0.95, 1.06)	0.841	1.00 (0.95, 1.05)	0.989	1.00 (0.95, 1.06)	0.922	1.00 (0.95, 1.06)	0.942

[1] composite of all-cause death, ACS, stroke, coronary artery revascularisation procedures

[2] composite of CVD death, ACS, stroke, coronary artery revascularisation procedures

[3] Model 1: Adjusted for sex and age

[4] Model 2: Model 1 plus additional adjustment for smoking, BMI, BP treatment, SBP, diabetes, cholesterol, HDL cholesterol, triglycerides, glucose, and CRP

[5] Model 3: Model 2 plus additional adjustment for COPD and eGFR

[6] Model 4: Model 3 plus additional adjustment for sCD14 and sCD163

ACS: acute coronary syndrome, BMI: body mass index, BP: blood pressure, COPD: chronic obstructive pulmonary disease, CRP: C-reactive protein, CVD: cardiovascular disease, eGFR: estimated glomerular filtration rate, HDL: high density lipoprotein, SBP: systolic blood pressure.
